# Provision of mother's own milk for preterm infants during the COVID-19 pandemic: Differential effect of insurance

**DOI:** 10.3389/fped.2022.1064931

**Published:** 2022-12-22

**Authors:** Lauren E. Boudreau, Betty R. Vohr, Richard Tucker, Elisabeth C. McGowan

**Affiliations:** ^1^ Department of Pediatrics, The Warren Alpert Medical School of Brown University, Providence, RI, United States; ^2^ Department of Pediatrics, Women & Infants Hospital of Rhode Island, Providence, RI, United States

**Keywords:** mother's own milk, neonatal intensive care unit (NICU), preterm (birth), coronavirus disease 2019 (COVID-19), pandemic, insurance, breast milk

## Abstract

Mother-infant dyads faced many challenges during the COVID-19 pandemic; however, the impact was different depending on socio-economic determinants. This study aims to investigate the impact of the COVID-19 pandemic on maternal provision of mother's own milk (MOM) at neonatal intensive care unit (NICU) discharge among preterm infants. We hypothesized that fewer infants would be discharged home on any MOM during the pandemic period compared to a pre-pandemic period. This is a retrospective analysis of infants born <34 weeks' gestation admitted to the Women and Infant's Hospital NICU. Infants born pre-pandemic (1/1/2019 to 2/29/2020) were compared to infants born during the pandemic (3/1/2020 to 4/30/2021). Maternal and neonatal variables were analyzed by group. The primary outcome was provision of MOM (defined as feeding exclusively MOM, or a combination of MOM and formula) at NICU discharge. Analyses were performed for time periods, and multivariable regression analyses were run for the total cohort and by insurance type. Analysis included 268 infants born pre-pandemic and 262 infants born during the pandemic. Pandemic group mothers vs. pre-pandemic were less likely to be single (27%, 63/233 vs. 38%, 93/243; *p* = 0.01) and more likely to have a diagnosis of chorioamnionitis (16%, 38/236 vs. 7%, 17/243; *p* = 0.002). Rates of public insurance were similar (55% pre-pandemic and 50% pandemic). There was no significant change in provision of MOM between time periods. In multivariable analysis, public insurance decreased the odds of MOM at discharge for the entire study period (aOR 0.31, 95% CI: 0.19–0.50; *p* = 0.0001). On analysis by insurance type, rates of MOM increased from 77% pre-pandemic to 88% during the pandemic (*p* = 0.03) for mothers with private insurance and remained unchanged for mothers with public insurance (52% pre-pandemic and 53% pandemic; *p* = 0.86). Mothers with private insurance had twice the odds (aOR 2.02, 95% CI: 1.02–3.97; *p* = 0.04) of providing MOM during the pandemic vs. pre-pandemic. For those with public insurance, the odds for any MOM provision during the pandemic were unchanged (aOR 0.95, 95% CI: 0.5–1.7; *p* = 0.86). These differences may be related to health care disparities requiring additional exploration of risk factors and the need for equitable opportunities for all mother-infant dyads.

## Introduction

Mother's own milk (MOM) is the ideal source of nutrition for infants, and in particular for infants born prematurely. MOM is beneficial in decreasing the risks of necrotizing enterocolitis, chronic lung disease, and late onset sepsis, and is associated with improved neurodevelopmental outcomes (1, 2). Yet despite these benefits, fewer preterm infants than term infant receive breast milk. In a recent report published by the Centers for Disease Control and Prevention, among infants born in the United States, 71.3% of preterm infants receive breast milk comparted to 84.6% of term infants (3). Unfortunately, there are known multi-factorial socioeconomic challenges such as poverty, low maternal education, and maternal race and ethnicity that are linked to decreased provision of MOM (4–8). Authors of a recent California cohort study reported a 52% lower odds of breast milk use at Neonatal Intensive Care Unit (NICU) discharge for families with public insurance compared to those with private insurance (8).

The onset of COVID-19 brought about unexpected and swift changes in the NICU environment. Early in the pandemic, NICU visitation policies were restrictive, limiting the number of parents who could visit as well as the frequency of visits. Such changes led to parental reported difficulties with breastfeeding, bringing in milk and supplies, communication and teaching moments, as well as overall decreased wellbeing (9–13). Additionally, many units experienced staffing changes that included scaled-down in-person lactation support (9, 14-16).

Currently, there are limited data published on the early impact of the pandemic on the provision of MOM for high-risk preterm infants, particularly between mothers with different insurance types. The primary aim of this study was to investigate maternal provision of MOM at the time of NICU discharge among preterm infants, and second, MOM provision for mothers with public vs. private insurance. Our primary hypothesis was that fewer infants would be discharged home on any MOM during the pandemic when compared to pre-pandemic.

## Methods

This is a single center, retrospective, observational study of preterm infants born <34 weeks gestational age (GA) at Women and Infant's Hospital (WIH) between January 2019 and April 2021 and who survived to NICU discharge. Exclusion criteria included infants with a congenital syndrome and infants who were transferred prior to discharge. The study protocol was approved by the WIH Institutional Review Board and informed consent was waived due to the retrospective nature of the study.

There were two comparison groups: infants born pre-pandemic (January 2019–February 2020), and infants born during the pandemic (March 2020-April 2021). March 2020 was selected as the start date of data collection for the pandemic group as per the World Health Organization (WHO) definition (17).

Maternal and infant data were collected from the electronic medical record and included the following variables: maternal age, marital status, insurance type (public vs. private), parity, multiple gestation, mode of delivery, presence of prenatal care, race, ethnicity, education level, and medical complications during pregnancy including maternal hypertensive disorders (inclusive of gestational hypertension, preeclampsia and eclampsia), gestational diabetes, placental abruption, and clinical chorioamnionitis. Infant variables included GA at birth, birth weight, sex, inborn status, discharge weight, length of hospital stay, and medical complications including sepsis (defined by a positive blood culture), bronchopulmonary dysplasia (defined as oxygen at 36 weeks), necrotizing enterocolitis (defined as Bell stage ≥2), and presence of a gastrostomy tube.

The primary outcome of this study was MOM at NICU discharge, which was defined as either feeding exclusively MOM or a combination of MOM and formula. All breast milk that infants received was MOM, as a donor human milk program was not in place at WIH during the study period.

### Lactation support services

The WIH lactation support program is an in-person service that is available seven days per week, eight hours per day, and this availability was unchanged during the pandemic time period. The lactation support team is comprised of three Certified Lactation Counselors (CLCs), including a native Spanish speaker, and two nurse International Board Certified Lactation Consultants (IBCLCs). The team assists all NICU mothers in three main areas: milk expression, preparing to breastfeed, and breastfeeding. Additionally, the lactation support team provides post-NICU support, including phone consultations and as needed outpatient lactation appointments. The IBCLCs create unit breastfeeding/lactation guidelines, lead the NICU Breastfeeding Committee, and provide education for the NICU staff.

### NICU visitation policy

The WIH NICU visitation policy was modified during the pandemic. The initial change restricted visitation to parents and grandparents only, with universal masking required. Within a week, visitation was further limited to two designated visitors per patient or set of multiples (typically the mother, and their partner/support person). The two designated visitors could be modified under extenuating circumstances, and exceptions were made, pending approval by hospital administration, for critically ill or dying infants. Each visitor was allowed only one visit per day of unlimited duration.

If a designated visitor had any symptoms of COVID-19, they were asked not to visit the hospital and to receive polymerase chain reaction (PCR) testing. If a designated visitor was exposed to someone positive for COVID-19, they were asked to quarantine for 10 days and a negative PCR test after quarantine was required to visit the hospital. If a designated visitor was positive for COVID-19, they were asked to quarantine for 10 days. If a baby was born to a mother positive for COVID-19, they could not visit the baby in the NICU for 10 days after symptom onset, or positive PCR test if asymptomatic. All cases of COVID-19 symptoms, exposure and positive testing were reviewed by the hospital pediatric infectious disease specialist who advised on testing and quarantine requirements.

### Statistical analysis

Maternal and infant characteristics were compared in bivariate analysis by using the *t* test or Wilcoxon test for continuous variables, and *χ*^2^ test for categorical variables. A multivariable logistic regression model was created to identify factors associated with the primary outcome of MOM at discharge. The model was adjusted for multiples, and pre-identified variables known to be confounders in the relationships of provision of MOM including maternal age, marital status, parity, race, education, and insurance status. Length of hospital stay was included to reflect infant health status. Models were run with clinical chorioamnionitis as it was significantly different between the time periods, however, was not retained as it did not contribute to the main regression model. Adjustment for multiple births was done using generalized estimating equations with an exchangeable correlation structure. Adjusted odds ratios were calculated with 95% confidence intervals. Secondary analyses were also conducted to study the relationship of insurance type and MOM at discharge. A *p*-value of <0.05 was considered statistically significant. All statistical analyses were conducted using SAS version 9.4 (SAS Institute).

## Results

A total of 479 mothers and 530 infants met inclusion criteria and were compared by pre-pandemic (*n* = 243 mothers and *n* = 268 infants) and pandemic (*n* = 236 mothers and *n* = 262 infants) time periods. Table 1 presents maternal characteristics. In the pandemic group, there were significantly fewer mothers who identified as single (27% vs. 38%, *p* = 0.01), and more cases of clinical chorioamnionitis (16% vs. 7%, *p* = 0.002) than pre-pandemic. Maternal social determinants of health including race, ethnicity, and education less than high school were similar between the two time periods. No differences were seen for public insurance pre-pandemic vs. pandemic (55% vs. 50%, *p* = 0.30). Table 2 presents infant characteristics. Infants born during the pandemic had a higher birth weight (1,481 g ± 528 g vs. 1,617 g ± 507 g, *p* = 0.005). This difference decreased but remained significant when controlling for GA (*p* = 0.02). Infant characteristics of medical complications, discharge weight, and length of NICU stay were similar between groups. For the primary outcome of any MOM at NICU discharge, analyzed by pre-pandemic vs. pandemic time periods, no significant differences (63% vs. 71%, *p* = 0.07) were seen for the total cohort. However, provision of any MOM at NICU discharge increased for mothers with private insurance during the pandemic (77% pre-pandemic vs. 88% pandemic, *p* = 0.03), while there was no change between time periods for mothers with public insurance (52% pre-pandemic vs. 53% pandemic, *p* = 0.86).

**Table 1:** Maternal characteristics and medical complications.

	Pre-Pandemic *n* (%)	Pandemic *n* (%)	*p* value
Maternal Characteristics	*n* = 243 (51%)	*n* = 236 (49%)
Maternal age, years (mean ± SD)	30 ± 6	30 ± 6	0.59
Single	93/243 (38)	63/233 (27)	0.01
Public Insurance	134/243 (55)	119/236 (50)	0.30
Primiparous	119/243 (49)	131/236 (56)	0.15
Multiple Birth	39/243 (16)	33/233 (14)	0.57
Cesarean Delivery	152/241 (63)	133/232 (57)	0.20
No Prenatal Care	4/240 (2)	3/233 (1)	0.73
Maternal Hypertensive Disorder	76/241 (32)	71/235 (30)	0.75
Gestational Diabetes	25/242 (10)	31/236 (13)	0.34
Placental Abruption	27/241 (11)	32/236 (14)	0.43
Chorioamnionitis	17/243 (7)	38/236 (16)	0.002
Non-White	94/242 (39)	89/236 (38)	0.80
Hispanic	59/241 (25)	46/234 (20)	0.21
Less than high school graduate	23/243 (9)	21/236 (9)	0.71

**Table 2:** Infant characteristics and medical complications.

	Pre-Pandemic *n* (%)	Pandemic *n* (%)	*p* value
Infant Characteristics	*n* = 268 (51%)	*n* = 262 (49%)
Gestational age at birth, mean (wks.) (mean ± SD)	30 ± 3	31 ± 3	0.08
Birth weight, mean (g) (mean ± SD)	1,481 ± 528	1,617 ± 507	0.005
Male sex	123/268 (46)	143/262 (55)	0.08
Outborn	16/267 (6)	16/261 (6)	0.95
Culture positive sepsis	12/268 (4)	10/261 (4)	0.70
Necrotizing Enterocolitis, proven	10/267 (4)	9/261 (3)	0.87
Gastrostomy Tube	23/268 (9)	17/262 (6)	0.48
Bronchopulmonary dysplasia, oxygen at 36 wk	46/267 (17)	39/260 (15)	0.50
Discharge weight, mean (g) (mean ± SD)	2,797 ± 926	2,757 ± 820	0.54
Length of stay in NICU, mean (days) (mean ± SD)	52 ± 43	46 ± 38	0.10
Any Human Milk at Discharge	170/268 (63)	186/262 (71)	0.07
Private Insurance	93/121 (77)	119/136 (88)	0.03
Public Insurance	77/147 (52)	67/126 (53)	0.86

Table 3 presents the multivariable regression models to predict any MOM at NICU discharge. For the total cohort, there was no effect of the pandemic on maternal provision of any MOM at NICU discharge (aOR 1.3, 95% CI: 0.84–1.95; *p* = 0.26). However, public insurance was an independent risk factor for lower odds of MOM provision (aOR 0.31, 95% CI: 0.19–0.50; *p* = 0.0001), and lower maternal education was associated with a 50% lower odds of MOM provision (aOR 0.5, 95% CI: 0.2–0.9; *p* = 0.03). In addition, for every 10 days in the NICU, there was a 13% decreased odds of MOM provision (aOR 0.87, 95% CI: 0.83–0.91; *p* = 0.0001).

**Table 3:** Logistic regressions of maternal & infant characteristics to predict MOM at discharge for the entire study period.

	Total Cohort *n* = 509	*p* value	Public *n* = 261	*p* value	Private *n* = 248	*p* value
	aOR (95% CI)		aOR (95% CI)		aOR (95% CI)	
Born during the pandemic	1.3 (0.84–1.95)	0.26	0.95 (0.5–1.7)	0.86	2.02 (1.02–3.97)	0.04
Maternal age, years	1.02 (0.98–1.06)	0.3	1.05 (1.0–1.1)	0.06	0.96 (0.9–1.0)	0.3
Public Insurance	0.31 (0.19–0.50)	0.0001	N/A	N/A	N/A	N/A
Single	0.7 (0.4–1.2)	0.17	0.95 (0.5–1.7)	0.87	0.4 (0.2–0.99)	0.048
Non-White Race	1.1 (0.71–1.71)	0.67	1.25 (0.7–2.1)	0.42	0.78 (0.4–1.7)	0.52
Less than HS graduate	0.5 (0.2–0.9)	0.03	0.4 (0.2–0.99)	0.047	0.4 (0.8–2.6)	0.37
Length of NICU Stay, 10 days	0.87 (0.83–0.91)	0.0001	0.82 (0.8–0.9)	0.0001	0.91 (0.84–0.98)	0.02

Separate regression models (Table 3) were estimated to predict any MOM at discharge by insurance type. During the pandemic, the odds of any MOM at discharge for mothers with public insurance remained unchanged (aOR 0.95, 95% CI: 0.5–1.7; *p* = 0.86), with a 18% decrease in odds of any MOM (aOR 0.82, 95% CI: 0.8–0.9; *p* = 0.0001) for every 10 additional days in the NICU. Mothers with private insurance had twice the odds of providing any MOM at NICU discharge (aOR 2.02, 95% CI: 1.02–3.97; *p* = 0.04), with a 9% decrease in odds of any MOM (aOR 0.91, 95% CI: 0.84–0.98; *p* = 0.02) for every 10 additional days in the NICU. Being single was associated with lower odds (aOR 0.4, 95% CI: 0.2–0.99; *p* = 0.048) of providing MOM at discharge for the private insurance group. Lower maternal education was marginally associated with lower odds (aOR 0.4, 95% CI: 0.2–0.99; *p* = 0.047) of providing MOM for the public insurance group only.

## Discussion

In this study, we report on the provision of MOM, comparing pre-pandemic and pandemic time periods for preterm infants discharged from the NICU. There was no association found between provision of MOM and the COVID-19 pandemic for the total cohort of preterm mother-infant dyads. However, after adjusting for covariates that predict MOM provision, a strong association was noted between private insurance and increased provision of MOM. These findings highlight a disparity between insurance types, especially as it relates to changes during the COVID-19 pandemic.

For our total cohort, maternal and infant characteristics and medical complications were similar between time periods, except for a few differences. There were more mothers with a clinical diagnosis of chorioamnionitis during the pandemic period. This finding is similar to the reports of other investigators. In a Canadian cohort, authors reported a 1.24 increased risk of clinical chorioamnionitis during the COVID-19 lockdown period compared to a corresponding 2015–2019 period (18). Despite the significant group difference identified in our data set, clinical chorioamnionitis did not have an impact on our primary outcome. However, several maternal medical complications during pregnancy that prevent early NICU visitation could potentially delay the initiation of expressing breast milk. In contrast, factors that enhance breast feeding, such as kangaroo care, could be explored further in the setting of pandemics (19).

An additional finding for our cohort was mothers were more likely to report being single during the pandemic. This may be related to COVID-19 isolation and social distancing guidelines, as marriages were often postponed during the pandemic (20). In our private insurance regression model, single status was associated with decreased odds of MOM provision at discharge. This finding may be partially explained by the importance of partner support for lactating mothers, particularly in times of overwhelming pandemic-related stress.

Another difference seen between time periods was infant birth weight, infants born during the pandemic were larger. There are conflicting reports on birthweight in pre-pandemic vs. pandemic time periods (21, 22). The magnitude of the difference decreased, though significance was retained when adjusting for GA. These findings may be a reflection that fewer infants were born at younger GA during the pandemic time period; the mean GA was one week greater in the pandemic time period. Although we interpret this finding with caution as GA differences were not statistically significant, Alshaikh et al. also report that GA and birth weight were higher in infants admitted to the NICU during the lockdown period (18).

While provision of MOM was similar between time periods, it was not until we explored rates by insurance type that clear group differences were identified. There was a significant increase in MOM for the private insurance group. A recent United Kingdom study by Hamid et al. that assessed poverty utilizing an index of multiple deprivation index (IMD) found similar results. Specifically that during the pandemic, women in the higher IMD (least deprived) quintiles were 2–4 times more likely to be feeding breast milk at NICU discharge than those in lower quintiles (23). It is possible that the mothers with private insurance have more opportunities to be present in the NICU, subsequently leading to increased exposure to supportive services and environments. Kelleher et al. reported patients with private insurance were able to spend more hours at the bedside per visit (5.7 h vs. 3.5 h), and an average of one more day per week in the NICU than patients with public insurance (24). Services such as in person lactation support, opportunities for skin-to-skin, participation in bedside care, and collaboration with the medical care team all facilitate successful breast milk production (25, 26). However, careful attention to not only the availability, but the accessibility of such services and opportunities for all groups of mothers is critical.

It was encouraging that provision of MOM did not significantly decrease for our mothers with public insurance. This may be a testament to mother's resiliency. Challenges that low-income mothers with public insurance may have faced during the pandemic include limited time off from work-place responsibilities, particularly if they were frontline or essential workers. In the United States, many essential workers, including those employed in public transit, nursing homes and food manufacturing plants, are reliant on public insurance (27, 28). Additional potential barriers low-income families may have encountered include limited access to transportation to the hospital, or problems finding care for children at home. NICU families with transportation difficulties are known to have decreased breast milk use at discharge (8). While data on maternal job status, available transportation, and home child care were not available for this cohort, they all relate to health care disparities and should be considered when supporting families of high-risk neonates.

Prolonged hospital stays provided a significant negative impact on MOM provision at discharge for our total cohort and separately for both insurance groups, which is not surprising as length of stay is a known risk factor for decreased MOM provision (29). One difficulty with maintaining breast milk provision is the amount of time needed for milk expression prior to each feed. For infants with prolonged hospital stay, mothers may need to return to work, challenging the ability to adequately express milk. Type of work has also been shown to have an impact on sustained provision of MOM which could relate to insurance status (30). Mothers in service/labor occupations have been reported to have the shortest breastfeeding duration (5.9 months average duration) as compared to non-working mothers or those in professional/managerial occupations (7.3–7.4 months average duration) (30). As longer hospital stays are correlated with illness severity among preterm infants, addressing disparities is critical.

### Strengths and limitations

Strengths of the study include a large cohort of high-risk preterm infants. To our knowledge, this is the first study in the United States to examine associations between the pandemic and provision of MOM for preterm infants and the differential effects of insurance type. Findings from our study are timely, as several pandemic-related policies are still in effect at hospitals, and may provide opportunity for modifications if needed. We recognize the limitations of this study, including the lack of data pertaining to confounders such as parental visitation, time spent with lactation team, and quantity of MOM available at discharge. Additionally, we did not collect data on maternal COVID positivity status, which may have impacted mother's ability to visit her neonate, and provide breast milk.

## Conclusion

Regression analysis of our total cohort identified the importance of the contribution of public health insurance to our outcome of MOM provision. In the separate adjusted regressions by insurance, rates of MOM provision for mothers with public insurance remained unchanged during the two time periods, whereas mothers with private insurance were twice as likely to provide MOM during the pandemic. In summary, during the pandemic, type of health insurance impacted on provision of MOM. The study findings can be leveraged to support the need for hospitals to continually monitor and evaluate outcomes to ensure equal and equitable opportunity for all families.

## Data Availability

The original contributions presented in the study are included in the article/supplementary material, further inquiries can be directed to Lauren Boudreau, lboudreau@wihri.org.
